# A Direct-Writing Approach for Fabrication of CNT/Paper-Based Piezoresistive Pressure Sensors for Airflow Sensing

**DOI:** 10.3390/mi12050504

**Published:** 2021-04-30

**Authors:** Jinyan Chen, Van-Thai Tran, Hejun Du, Junshan Wang, Chao Chen

**Affiliations:** 1 State Key Laboratory of Mechanics and Control of Mechanical Structures, Nanjing University of Aeronautics and Astronautics, Nanjing 210016, China; jychen@nuaa.edu.cn (J.C.); wjs@nuaa.edu.cn (J.W.); 2 School of Mechanical and Aerospace Engineering, Nanyang Technological University, 50 Nanyang Avenue, Singapore 639798, Singapore; vanthai.tran@ntu.edu.sg (V.-T.T.); MHDU@ntu.edu.sg (H.D.)

**Keywords:** piezoresistive, carbon nanotubes, direct write, paper, airflow

## Abstract

Airflow sensor is a crucial component for monitoring environmental airflow conditions in many engineering fields, especially in the field of aerospace engineering. However, conventional airflow sensors have been suffering from issues such as complexity and bulk in structures, high cost in fabrication and maintenance, and low stability and durability. In this work, we developed a facile direct-writing method for fabricating a low-cost piezoresistive element aiming at high-performance airflow sensing, in which a commercial pen was utilized to drop solutions of single-walled carbon nanotubes onto tissue paper to form a piezoresistive sensing element. The encapsulated piezoresistive element was tested for electromechanical properties under two loading modes: one loading mode is the so-called pressure mode in which the piezoresistive element is pressed by a normal pressure, and another mode is the so-called bending mode in which the piezoresistive element is bended as a cantilever beam. Unlike many other developed airflow sensors among which the sensing elements are normally employed as cantilever beams for facing winds, we designed a fin structure to be incorporated with the piezoresistive element for airflow sensing; the main function of the fin is to face winds instead of the piezoresistive element, and subsequently transfer and enlarge the airflow pressure to the piezoresistive element for the normal pressure loading mode. With this design, the piezoresistive element can also be protected by avoiding experiencing large strains and direct contact with external airflows so that the stability and durability of the sensor can be maintained. Moreover, we experimentally found that the performance parameters of the airflow sensor could be effectively tuned by varying the size of the fin structure. When the fin sizes of the airflow sensors were 20 mm, 30 mm, and 40 mm, the detection limits and sensitivities of the fabricated airflow sensors were measured as 8.2 m/s, 6.2 m/s, 3.2 m/s, 0.0121 (m/s)^−2^, 0.01657 (m/s)^−2^, and 0.02264 (m/s)^−2^, respectively. Therefore, the design of the fin structure could pave an easy way for adjusting the sensor performance without changing the sensor itself toward different application scenarios.

## 1. Introduction

Airflow sensors play significant roles in many engineering fields including environmental monitoring, sustainable energy utilization, aerospace engineering and so forth. For instance, the maneuverability and stability of a flying aircraft highly relies on the real-time monitoring and perception of the surrounding airflow conditions [1,2,3,4,5,6,7]. Moreover, the small size and light weight of unmanned aerial vehicles (UAVs) and micro air vehicles (MAVs) make them susceptible to impact caused by their surrounding airflows [3,4]. Therefore, monitoring of the surrounding airflow is crucial because it contains the necessary information for controlling air vehicles [5,6,7]. Traditional anemometers mostly adopt mechanical rotating structures for airflow sensing, which are structurally bulky and complicated and require high manufacturing accuracy. With the advancement of novel materials and techniques, researchers have devoted their efforts into developing various types of airflow sensors based on diversified sensing mechanisms such as thermal flow sensors [5,7,8], ultrasonic wind sensors [9,10], and vortex flow meters [11]. However, these types of airflow sensors suffer from some common issues including high cost of fabrication, drift problem during operation [12,13], large size and weight [14,15], and poor accuracy [16,17]. Thanks to the emergence of smart materials and devices over recent years [18,19], the current mainstream airflow sensors mainly rely on three types of sensing principles (i.e., piezoresistive [19,20,21], piezoelectric [1,22,23], and capacitive [24] principles). Compared with piezoresistive airflow sensors, capacitive airflow sensors mainly suffer from slow responses [16,24,25]; piezoelectric airflow sensors have little capability in sensing dynamic airflows, which limits their application ranges [1,22]. Furthermore, both capacitive and piezoelectric airflow sensors require complicated measurement circuits and signal processing methods [1]. These disadvantages make piezoresistive airflow sensors more attractive in some applications due to their simple and compact structures, low cost, low power consumption, and convenience of signal acquisition and processing [26].

To develop high-performance piezoresistive sensing elements, researchers have been focusing on advanced functional nanomaterials, fabrication techniques, substrate types, and so on. Among these well-developed nanomaterials, zero-dimensional and one-dimensional metallic nanomaterials, carbon nanotubes (CNTs) and graphene are the most commonly utilized nanomaterials for constructing decent piezoresistive sensing elements due to their outstanding electromechanical properties. Another significant task for researchers to obtain desirable piezoresistive sensing elements is to explore versatile fabrication methods for sensing films. Spin-coating [27], dip-coating [28], spray-coating [29], vacuum filtration [30], and inkjet printing [31] are the current mainstream solution deposition techniques to prepare piezoresistive sensing films. In general, these methods require professional users to perform fabrication processes and operate the related fabrication equipment, which may not be friendly for simple and large-scale device fabrications. Thus, it is highly desired that more facile and reliable fabrication techniques for piezoresistive sensors are proposed and investigated.

In this work, we adopted a facile strategy to prepare piezoresistive sensing elements and propose a novel sensor configuration for the subsequent airflow detection applications. Through writing a high-purity single-walled CNT (SWCNT) aqueous dispersion directly onto tissue paper via a fountain pen to form a sensing element, the sensing element can be fabricated in a simple and low-cost manner. Control of size accuracy can be implemented by changing fountain pens with different pen nib sizes, which could be promising for the miniaturization of the sensor. Additionally, through fountain pen-enabled controllable direct writing sensing elements, the performance consistency among the fabricated elements can be improved compared to other common fabrication techniques such as drop casting. The encapsulated piezoresistive element was tested for electromechanical properties under the pressure loading mode and the bending loading mode. In order to solve the issues of over-strain and instability of airflow sensors experiencing bending, we employed the pressure loading mode other than the bending loading mode of the sensing element for airflow detection. With this in mind, we employed a novel fin cantilever structure to be integrated with the piezoresistive element for airflow sensing, in which the fin structure is utilized to convert and amplify the airflow dynamic pressure into the pressure exerted on the piezoresistive sensor. Thanks to this design, the piezoresistive sensor can experience less strain, which is conducive to the stability and wider working range of the sensor. Through experimentation, we found that the performance parameters of the piezoresistive airflow sensor can be adjusted by changing the size of the fin structure. Furthermore, thanks to the employment of the piezoresistive mechanism, the sensor measurement system has a simple signal acquisition circuit without the usage of aa signal amplifier, which could be beneficial to the miniaturization and integration of the airflow sensor. This study could pave the way for further research on the fabrication and utilization of piezoresistive airflow sensing elements experiencing pressure loading mode for air monitoring devices in assorted applications such as wind-driven generators, UAVs, and MAVs. 

## 2. Methodology

### 2.1. Fabrication of the Piezoresistive Elements

**Figure 1:**
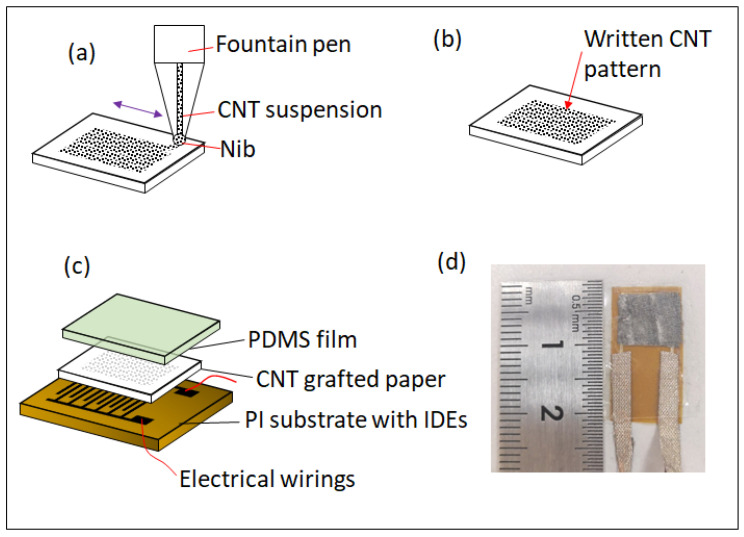
(**a**) Direct writing CNT suspensions onto tissue paper. (**b**) Paper with written CNT patterns. (**c**) Assembling a piezoresistive element. (**d**) Photo of a fabricated piezoresistive element.

The fabrication process of the piezoresistive element is described in Figure 1, in which the piezoresistive element is composed of three layers from top to bottom: PDMS film, CNT-grafted paper, and a polyimide (PI) substrate with interdigital electrodes (IDEs). Specifically, elastic PDMS film was prepared by mixing silicone gel (Sylgard 184, Dow Corning) with a curing agent with a mass ratio of 10:1, stirring, and vacuuming for removal of air bubbles. The mixture was spin-coated on a glass substrate to form a thin film with a thickness of about 100 µm. Then, it was cured at 60 °C for 8 h. The film was then cut into pieces with a size of 10 mm by 20 mm. The PI substrate with IDEs was custom-made with the specific parameters as follows: the overall size was 10 mm × 20 mm; the thickness was 13 µm; the finger width was 100 µm; the finger distance was 100 µm; the finger length was 6.3 mm; and the number of finger pairs was 20. The conductive layer was constructed by stacking thin films of copper, nickel, and gold with thicknesses of 12 µm, 3 µm, and 1 µm, respectively.

In the fabrication scheme of the piezoresistive element, the most critical process is direct writing of the CNT solutions onto the tissue paper, as shown in Figure 1a. In this process, the first step is the preparation of the CNT solution. A high-purity CNT aqueous dispersion with the SWCNT concentration of 0.2 wt% was purchased from Chengdu Organic Chemical Corporation. The CNT aqueous dispersion was loaded into the fountain pen with a nib and ready for usage. Upon the nib touching the tissue paper, due to the porous structure of the paper’s cellulose fiber, which facilitates the capillary effect, the liquid tended to be pulled from the nib to the paper, which resulted in the deposition of the CNT suspension onto the tissue paper. In order to fully prepare the sensing film, the fountain pen was used to draw a square pattern with the size of 8 mm × 8 mm onto the tissue paper. The paper was then dried at 60 °C to facilitate the evaporation of water, and the CNTs adhered to the paper due to the weak hydrogen bonding effect and the CNT-grafted pattern on the paper was formed, as shown in Figure 1b.

The last step in the fabrication process is encapsulation. The CNT-grafted tissue paper was flipped over so that the paper could face the IDEs to form a contacting interface. Two metal wires were bonded to the pads of the IDEs using conductive fabric tapes for the electrical connection. Then, a prepared PDMS film was used to cover the CNT-grafted tissue paper and PI substrate for encapsulation, as shown in Figure 1c. Figure 1d shows a photo of the assembled piezoresistive element, the resistance of which was measured to be about 5.3 kΩ.

### 2.2. Measurement and Characterization

**Figure 2:**
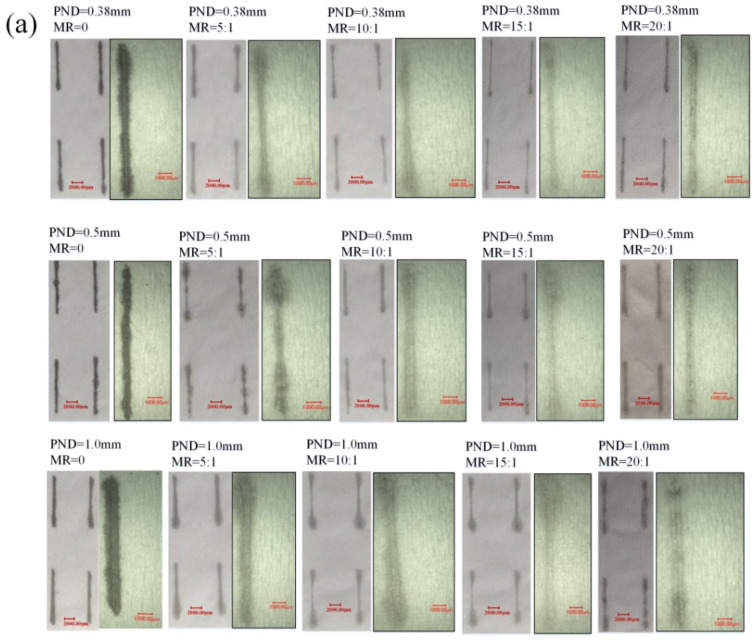

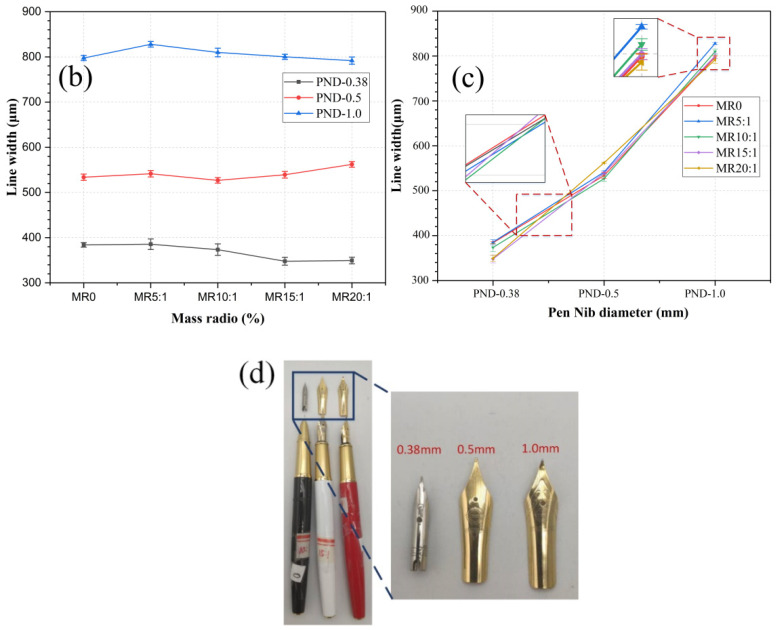
(**a**) Images of the written lines using different nib sizes and CNT suspension concentrations. (**b**) Measured widths of the written lines versus MR under different PNDs. (**c**) Relationship between the width of the written line and PND under different MRs. (**d**) Fountain pens with nib sizes of 0.38 mm, 0.5 mm, and 1.0 mm, respectively.

Images of written CNT lines using fountain pens with different nib sizes and different CNT suspension concentrations were taken by an optical microscope and are shown in Figure 2. The widths of the written lines were measured and analyzed using ImageJ software. The dimensional precision of the drawn patterns was investigated by comparing the line widths, and the details are shown in Figure 2a. The influences of the concentration of CNT suspension (i.e., the mass ratio (MR)), and the pen nib diameter (PND) on the patterned line widths are shown in Figure 2b–d. It can be seen that there was no obvious change in the line width of the drawn pattern when the MR varied from 0 to 20:1. When the nib sizes were 0.38 mm, 0.5 mm, and 1.0 mm, the corresponding line widths were about 300 µm, 540 µm, and 800 µm, respectively. Aside from the increasing trend, it was seen that the line width values were approximately equal to the values of the PND. Therefore, the line width of the drawn pattern can be determined and tuned by nib size under normal manual writing speeds. The capability of achieving a high-precision size of the written pattern provides evidence that the direct writing method is capable of creating small sized patterns by using a small pen nib, which is essential to facilitate device miniaturization.

**Figure 3:**
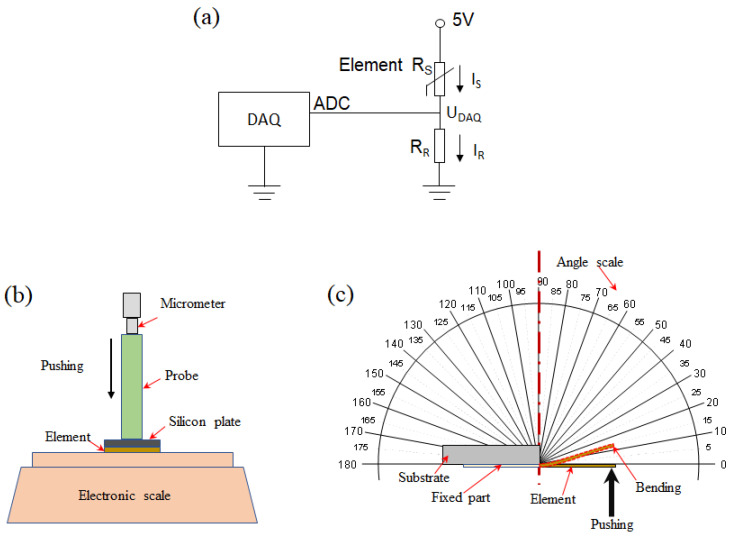
(**a**) Schematic of the electrical measurement circuit. (**b**) Pressure loading mode for the piezoresistive element. (**c**) Bending loading mode for the piezoresistive element.

The main characteristics of the fabricated piezoresistive element are the resistance changes under external loadings. A one-kilo Ohms resistor was connected in series with the element to create a voltage divider circuit. A NI data acquisition (DAQ) card was utilized to record the voltage change caused by the variation of the element’s resistance, and a 5 V base voltage was provided for the circuit by the digital pin of the DAQ card. During the testing process, the sampling frequency was set at 1 kHz and the digital signals of response voltage could be monitored from the computer. The circuit diagram is shown in Figure 3a.

The current running through the circuit was identical and can be determined using Ohm’s law:

(1) $$I_{R}=I_{S}=I$$

(2) $$I_{R}=\frac{U_{R}}{R_{R}}=\frac{U_{DAQ}}{R_{R}}$$

(3) $$I_{S}=\frac{U_{S}}{R_{S}}$$

where *R_R_* and *R_S_* are the resistances of the in-series resistor and the piezoresistive element, respectively; *I_R_* and *I_S_* are the currents running through the in-series resistor and the piezoresistive element, respectively; and *U_R_* and *U_S_* are the voltages across the in-series resistor and the piezoresistive element, respectively. *I* is the current running through the circuit and is identical with *I_R_* and *I_S_*. *U_DAQ_* is the voltage measured by the DAQ card, and is identical with *U_R_*.

The current change of the piezoresistive element was the same as that of the in-series resistor. The variation trend of the current in the circuit (i.e., (*I* − *I*_0_)/*I*_0_) can be determined by

(4) $$\frac{I-I_{0}}{I_{0}}=\frac{\frac{U_{DAQ}}{R_{R}}-\frac{U_{0}}{R_{R}}}{\frac{U_{0}}{R_{R}}}=\frac{U_{DAQ}-U_{0}}{U_{0}}$$

where *I*_0_ and *U_0_* are the original current in the circuit and voltage of the resistor without external loading.

The electromechanical characteristics of the piezoresistive element were studied under two loading modes: pressure loading mode and bending loading mode, as shown in Figure 3b,c respectively. In the pressure loading mode, the resistance of the piezoresistive element was measured under different normal pressures. The element was put on an electronic scale (LQ-C8002, Shanghai Yaoxin Electronic Technology Co. Ltd., Shanghai, China). The pressure was generated and adjusted using a micrometer, which pushes the element via a square plate with the size of 8 mm × 8 mm to create an even pressure to the element. The electronic scale recorded the applied pressure value in term of mass (i.e., the applied mass varying from 0 to 270 g corresponding to 0 to 42 kPa in pressure). With the increase in applied pressure, the resistance of the element was reduced, leading to a rise in electrical current. As shown in Figure 3b, since the pressure exerted on the element could be obtained by dividing the applied force to the area of the square plate, the sensitivity of the element in the pressure mode can be determined by the formula as *S = (*Δ*I/I*_0_*)/*Δ*P* [32]. In the bending loading mode, the cantilever of the element was manually bent to a specific angle following a printed angle scale, as shown in Figure 3c. An angle scale and the NI DAQ card recorded the bending angle and the electric signal, respectively. Considering practical application scenarios, the bending mode test was conducted with the bending angle in the range of 0 to 90 °C degrees. With the increase in applied bending angle, the deformation as well as strain of the element increased; hence the resistance of the element was reduced, leading to the increase in the current (transduced by Ohm’s law). Therefore, the sensitivity of the element in the bending mode was found to be *S = (*Δ*I/I*_0_*)/α*, where α is the bending angle of the element.

### 2.3. Experimental Setup for Airflow Sensing

**Figure 4:**
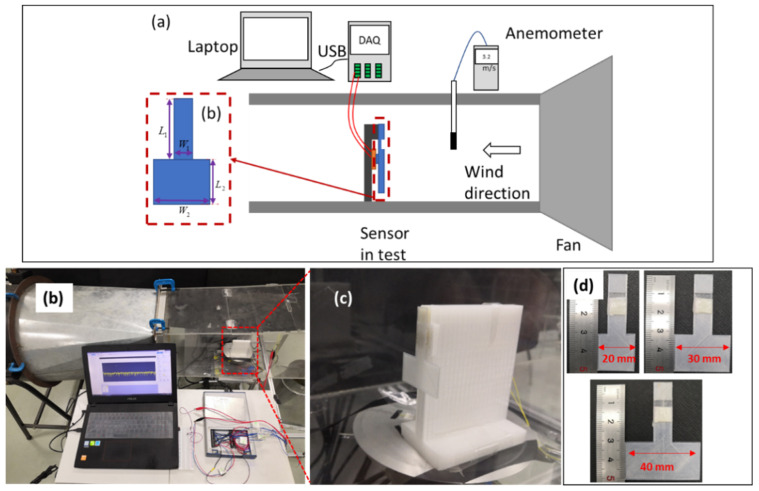
(**a**) Schematic of the experimental setup for airflow sensing. (**b**) Photo of the setup. (**c**) Piezoresistive airflow sensor inside a 3D-printed holder. (**d**) Different fin sizes for experimentation.

In order to characterize the airflow sensing performance of the fabricated piezoresistive element, we set up an experimental system for the test. Figure 4a,b shows a schematic and a photo of the airflow sensing test system, respectively. The test platform was composed of a small wind tunnel, an anemometer (Smart Sensor Co., Ltd., Hongkong, China) a NI DAQ card, a computer, and a piezoresistive airflow sensor. The airflow velocity was manually adjusted, and the anemometer was employed for calibration of the wind speed.

In this setup, unlike conventional airflow sensors, which commonly use the cantilever beam structures of the sensing elements to receive the airflow loadings, we proposed a fin structure integrated with the piezoresistive element to leverage the pressure loading mode for the airflow sensing functionality. Since the area of the front face of the fin is much larger than that of the back face of the fin, the actual airflow pressure exerted on the element is much larger than the inlet airflow pressure. In this regard, the sensitivity of the piezoresistive element can be enhanced through the introduction of the fin structure and the sensitivity can also be tuned by using fins with different areas of front faces. Moreover, the usage of the fin can avoid direct contact between the element and external airflows and large deformation of the sensing element, both of which are conducive to the stability and durability of the airflow sensor. A set of fins with sizes of 20 mm, 30 mm, and 40 mm were printed using a 3D printer and polylactic acid (PLA) material, respectively, as shown in Figure 4d. As shown in Figure 4b,c, the fin was set in aa perpendicular direction with the airflow, and the airflow sensor including the piezoresistive element and the fin was mounted on a 3D printed holder to keep the sensor in the middle of the airflow in the wind tunnel.

## 3. Characteristics and Discussion

### 3.1. Electromechanical Properties of the Piezoresistive Element under Two Loading Modes

#### 3.1.1. Pressure Mode

**Figure 5:**
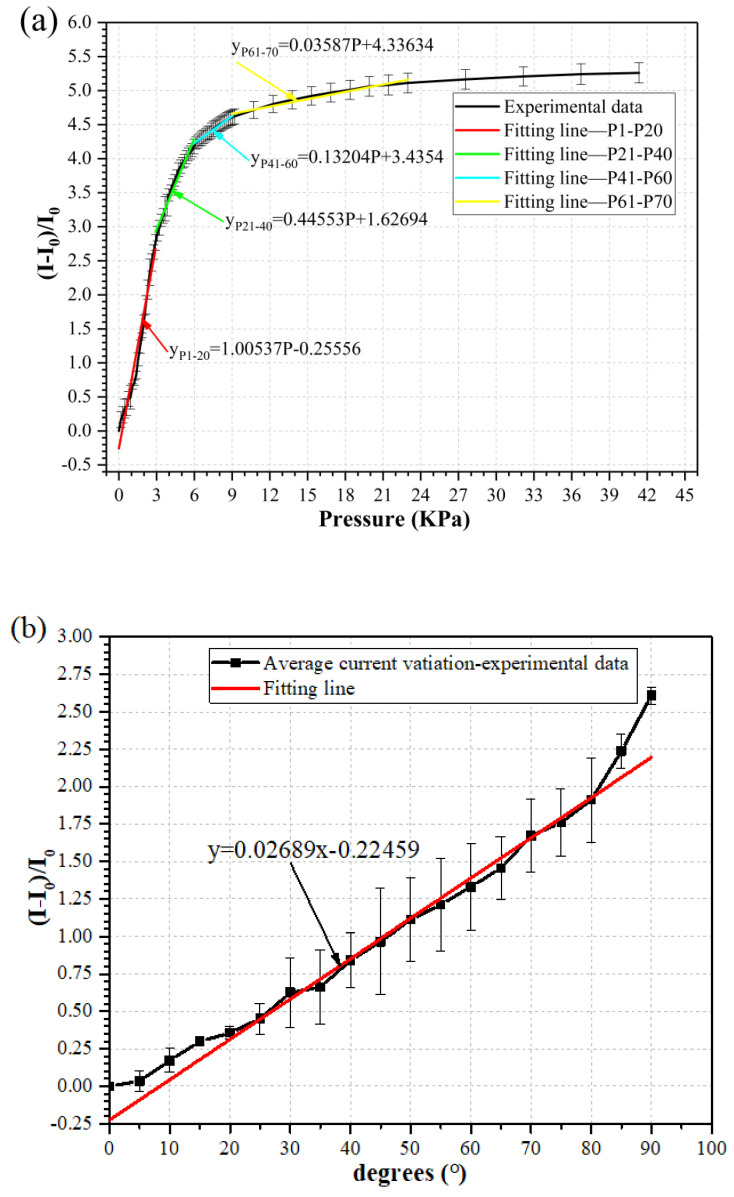
Electromechanical properties of the piezoresistive element under (**a**) pressure mode; (**b**) bending mode.

As shown in Figure 5a, with the increase in applied pressure, the resistance of the piezoresistive element was reduced, leading to a rise in the electrical current, and the element could produce a response at a pressure value as low as 153 Pa, which corresponded to a mass of 1 g. The response can be divided into multiple linear regions similar to previous reports [32,33], and subsequently, the fitting curves can be divided into four sections. In the small pressure range from 0 kPa to 3 kPa, the response of the element was linear, and fitted to the red line. The sensitivity of the element in this range was calculated to be 1.00537 kPa^−1^, which is the slope of the fitting curve. In the larger pressure range from 3 kPa to 23 kPa, the sensitivity was smaller than that in the first region such as 0.44 kPa^−1^, 0.13 kPa^−1^, and 0.035 kPa^−1^ for the second, the third, and the fourth regions, respectively. Our wind sensor working range is supposed to be in the small pressure region due to the small wind speed, so the sensor will likely work in the first linear region of pressure response.

#### 3.1.2. Bending Mode

It can be seen from Figure 5b that in the bending loading mode, when the loading angle changed from 0° to 90°, the response of the sensor element could be linearly fitted, and the sensitivity was calculated to be 0.02689 degree^−1^. Within the bending angle ranging from 0° to 90°, the current change of the sensor element was smaller than that in the range of 0 to 23 kPa in the pressure loading mode.

#### 3.1.3. Comparing the Two Modes

Since airflows can only create small pressures to the sensing element, the detection limits of the sensor element under two loading modes need to be compared to choose a suitable working mode. For the bending mode, the detection limit is about 5° since the corresponding current variation value is very small. Therefore, a deformation angle larger than 5° is required to be exerted on the sensor element to give a considerable output signal under the bending mode. For the pressure mode, the detection limit is as low as 153 Pa. It is seen from Figure 5a,b that the current variations ranged from 0 to 5.2 under the pressure mode and from 0 to 2.6 under the bending mode, respectively, which implies that the sensor element may exhibit a larger sensitivity and wider working range under the pressure loading mode. Furthermore, the piezoresistive element under the pressure loading mode could withstand a pressure of up to 42 kPa repeatedly, while the upper limit of the sensor element in the linear range under the bending mode was limited by 90°.

### 3.2. Airflow Sensing Characteristics

#### 3.2.1. Raw Data and Signal Processing Method

**Figure 6:**
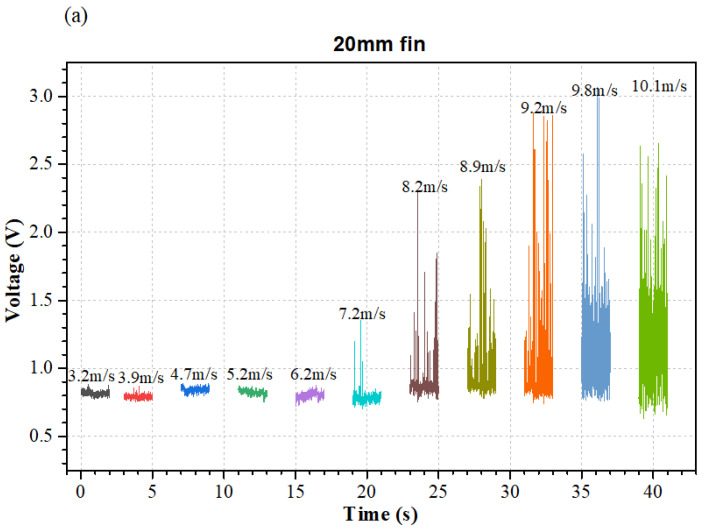

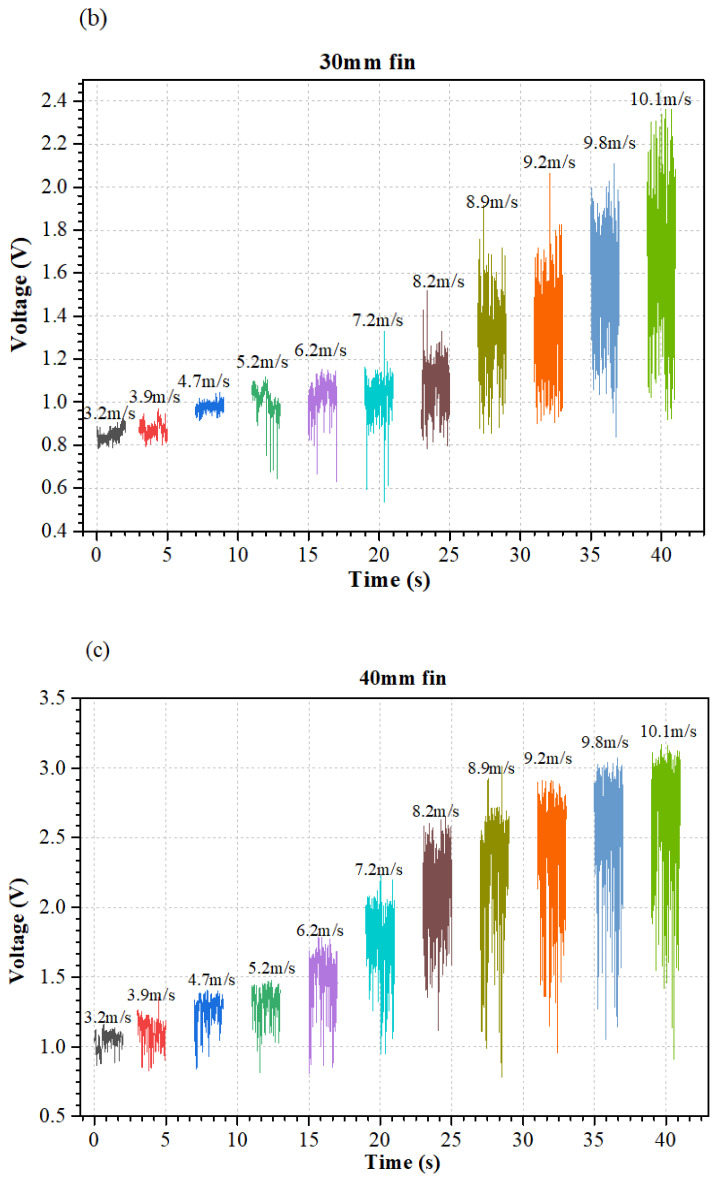
Raw voltage signals of the airflow sensors with fin sizes of (**a**) 20 mm; (**b**) 30 mm; (**c**) 40 mm.

Figure 6a–c presents the raw voltage signals of the airflow sensors with 20 mm fin, 30-mm fin, and 40-mm fin, respectively. It was seen that as the airflow velocity increased in the test range, the voltage signal exhibited an increasing trend. However, it was noticed that the maximum voltage was obviously unstable. Therefore, the maximum voltage value cannot be effectively utilized for evaluating the relationship between the measured voltage and airflow velocity.

**Figure 7:**
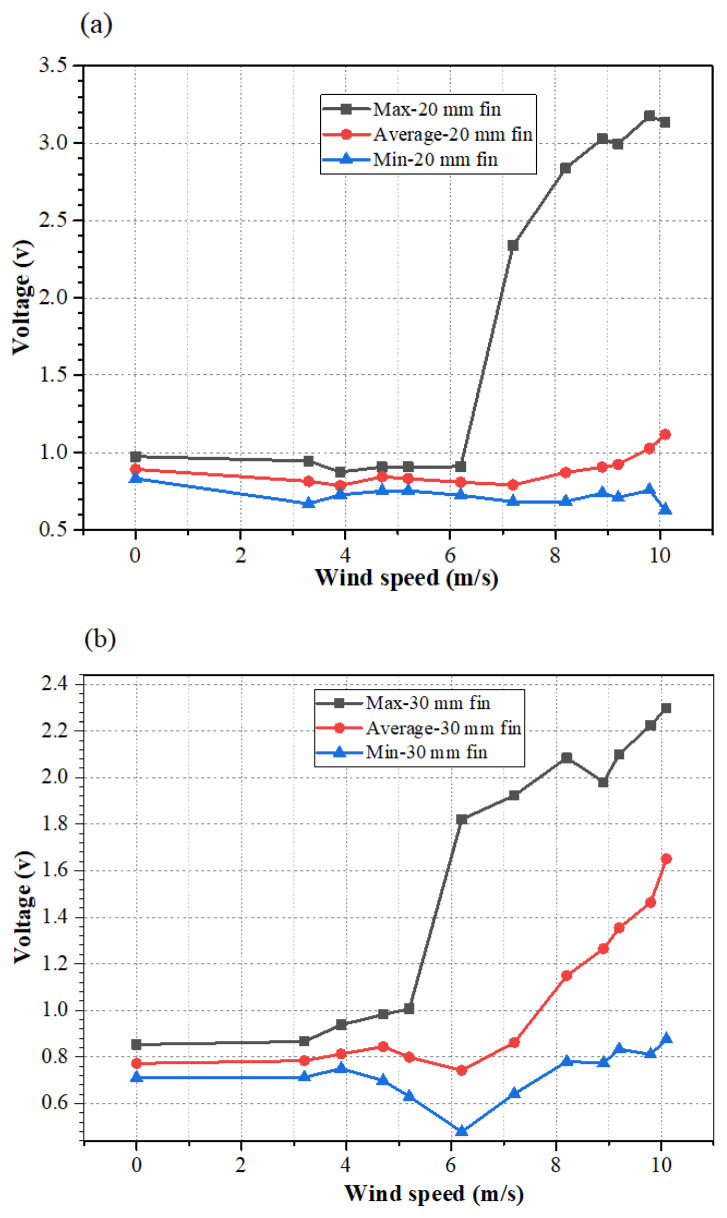

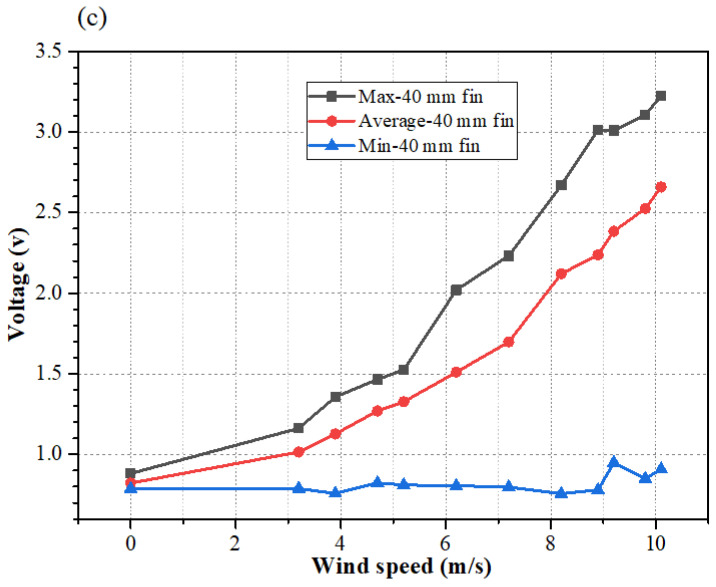
Maximum, minimum, and average values of voltage signals versus wind speed under fin sizes of (**a**) 20 mm; (**b**) 30 mm; (**c**) 40 mm.

Figure 7 shows the extracted voltage signals using maximum, minimum, and average values of the measured data. It can be seen that the maximum and minimum values were heavily affected by noise, which was similar to the observation in the raw data in Figure 6. Therefore, these data were not suitable to be used as the characteristic signal for the sensor. However, the average value of acquired voltage signal could be used for the calibration of airflow velocity. This is because this signal is less affected by noise, and its increasing trend is well consistent with the increment of the airflow velocity.

#### 3.2.2. Curve Fitting

The function *f(x) = kx*^2^ *+ b* was utilized to fit the data from the measured current variations in the circuit at different wind speeds, and the fitting results are shown in Table 1. This approach was also adopted by other studies [16,34]. While the response of the 40 mm fin showed good agreement with the fitting curve, the data from other fins with different sizes did not fit well with the function, in terms of the coefficient of determination (COD) R-squared values were smaller than 0.95, as shown in Table 1. This is because sensors with fin sizes of 20 mm and 30 mm cannot work in a low airflow speed range since the piezoresistive element experiences very small pressure.

**Figure 8:**
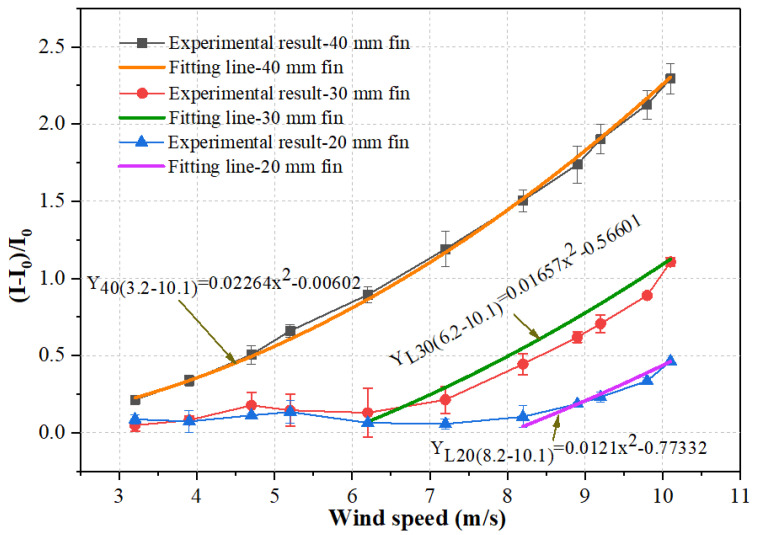
Relationships between the current variations and wind speed under different fin sizes.

Local fittings are required for the 20 mm fin and 30 mm fin sensor in their working ranges. For these sensors with 20 mm fin and 30 mm fin, the above fitting function can still be utilized for fitting local data points, as shown in Figure 8. Fitting range was selected using a trial-and-error method, until a good fitting was obtained based on the evaluation of the R-squared values. For the 20 mm fin, the obtained fitting curve was *Y_L20(V8.1–V10.1)_ =* 0.0121*v*^2^ − 0.77332 with an R^2^ value of 0.99362 in the working range from 8.2 m/s to 10.1 m/s. Similarly, for data obtained by the 30 mm fin sensor, in the working range from 6.2 m/s to 10.1 m/s, the fitting curve was *Y _L30(V6.2–V10.1)_ =* 0.01657*v*^2^ − 0.56601 with an R^2^ value of 0.97671. Thus, it is generally confirmed that the current variation of the sensor linearly follows the variation of the square of wind speed.

#### 3.2.3. Detection Limit

Figure 8 shows the current variations versus airflow velocity under different fin sizes. Combined with the above curve fitting discussion, it can be found that the detection limits of airflow velocity were 8.2 m/s, 6.2 m/s, and 3.2 m/s when the fin sizes were 20 mm, 30 mm, and 40 mm, respectively. This result demonstrates that using a larger fin size is beneficial in obtaining a lower detection limit since a larger fin size can enlarge more airflow pressure to the sensing element. Although the detection limit was 3.2 m/s when the fin size was 40 mm, this value was limited by the capability of our in-hand wind tunnel (the smallest airflow velocity can be generated is 3.2 m/s), which means that the detection limit of the airflow sensor may be even lower than 3.2 m/s. The above presented experimental results indicate that the detection limit of the piezoresistive airflow sensor can be effectively tuned by using the proposed fin structures with different sizes.

#### 3.2.4. Sensitivity

As shown in Figure 8, the current variation linearly followed the second power of airflow velocity. Thus, the sensitivity of the airflow sensor (i.e., (I-I_0_)/∆v^2^) can be obtained as the linear slope of the plot before the sensor exhibits current saturation. Moreover, the sensitivity can also be determined with the fitting parameter k in Table 1 [26,32]. It was seen that the airflow sensor with a larger fin size held larger sensitivity. In particular, the sensitivities of the airflow sensors were 0.0121 (m/s)^−1^, 0.01657 (m/s)^−1^, and 0.02264 (m/s)^−1^ when the fin sizes were 20 mm, 30 mm, and 40 mm, respectively. This sensitivity value was larger than the recent reported resistance-type airflow sensors such as a MEMS-based airflow sensor with sensitivity from 1.79 × 10^−4^ (m/s)^−1^ to 8.07 × 10^−4^ (m/s)^−1^ and an ultra-thin piezoresistive silicon cantilever airflow sensor with sensitivity from 0.65 × 10^−5^ (m/s)^−1^ to 4.49 × 10^−5^ (m/s)^−1^ [35,36].

## 4. Conclusions

In this paper, we demonstrated a facile direct-writing method to fabricate a piezoresistive airflow sensor using the pressure loading mode of the sensor, in which a fountain pen with CNT suspension was used to graft CNT onto tissue paper to form a piezoresistive sensing element. The packaged piezoresistive element was tested for its electromechanical properties under the pressure loading mode and the bending loading mode. We proposed a fin structure incorporated with the piezoresistive element to realize high-performance airflow sensing such as a low detection limit of 3.2 m/s and high sensitivity of 0.02264 (m/s)^−2^. It was also shown that the current variation of the sensor had a proportional relationship with the square of the wind speed. Through experimentation, we found that the performance parameters of the airflow sensor could be effectively adjusted by varying the size of the fin structure. The demonstrated fabrication method is suitable for the large-scale fabrication of devices and the designed fin structure is capable of tuning the sensor’s performance without changing the sensor itself. We believe that the presented results in this paper are of great potential for the facile fabrication of nanomaterial-based functional devices and could move forward the investigation of airflow sensing devices.

## Figures and Tables

**Table 1:** Fitting parameters for different fins.

Fitting Function	k	b	R-Squared (COD)	Adj. R-Squared
*Y_40(3.2–10.1)_*	0.02264	−0.00602	0.99801	0.99779
*Y_30(3.2–10.1)_*	0.01009	−0.06366	0.90176	0.89084
*Y_L30(V5.2–V10.1)_*	0.01318	−0.26525	0.9148	0.90059
*Y_L30(V6.2–V10.1)_*	0.01657	−0.56601	0.97671	0.97205
*Y_20(3.2–10.1)_*	0.00463	−0.08348	0.77769	0.75299
*Y_L20(V6.2–V10.1)_*	0.00591	−0.19474	0.86661	0.83994
*Y_L20(V7.2–V10.1)_*	0.01131	−0.69888	0.96796	0.95995
*Y_L20(V8.2–V10.1)_*	0.0121	−0.77332	0.99362	0.9915

## Data Availability

Not applicable.
